# Mirtazapine Inhibits Tumor Growth via Immune Response and Serotonergic System

**DOI:** 10.1371/journal.pone.0038886

**Published:** 2012-07-13

**Authors:** Chun-Kai Fang, Hong-Wen Chen, I-Tsang Chiang, Chia-Chieh Chen, Jyh-Fei Liao, Ton-Ping Su, Chieh-Yin Tung, Yosuke Uchitomi, Jeng-Jong Hwang

**Affiliations:** 1 Department of Biomedical Imaging and Radiological Sciences, National Yang-Ming University, Taipei, Taiwan; 2 Department of Psychiatry and Suicide Prevention Center, Mackay Memorial Hospital, Taipei, Taiwan; 3 Department of Radiation Oncology and Hospice Palliative Care Center, Mackay Memorial Hospital, Taipei, Taiwan; 4 Institute of Nuclear Energy Research, Taoyuan, Taiwan; 5 Institute of Pharmacology, National Yang-Ming University, Taipei, Taiwan; 6 Department of Psychiatry, Taipei Veterans General Hospital, Taipei, Taiwan; 7 Department of Neuropsychiatry, School of Medicine, Dentistry and Pharmaceutical Sciences, Okayama University, Okayama-shi, Japan; IIT Research Institute, United States of America

## Abstract

To study the tumor inhibition effect of mirtazapine, a drug for patients with depression, CT26/*luc* colon carcinoma-bearing animal model was used. BALB/c mice were randomly divided into six groups: two groups without tumors, i.e. *wild-type* (no drug) and *drug* (mirtazapine), and four groups with tumors, i.e. *never* (no drug), *always* (pre-drug, i.e. drug treatment before tumor inoculation and throughout the experiment), *concurrent* (simultaneously tumor inoculation and drug treatment throughout the experiment), and *after* (post-drug, i.e. drug treatment after tumor inoculation and throughout the experiment). The “psychiatric” conditions of mice were observed from the immobility time with tail suspension and spontaneous motor activity post tumor inoculation. Significant increase of serum interlukin-12 (sIL-12) and the inhibition of tumor growth were found in mirtazapine-treated mice (*always*, *concurrent*, and *after*) as compared with that of *never*. In addition, interferon-γ level and immunocompetent infiltrating CD4+/CD8+ T cells in the tumors of mirtazapine-treated, tumor-bearing mice were significantly higher as compared with that of *never.* Tumor necrosis factor-α (TNF-α) expressions, on the contrary, are decreased in the mirtazapine-treated, tumor-bearing mice as compared with that of *never*. Ex vivo autoradiography with [^123^I]ADAM, a radiopharmaceutical for serotonin transporter, also confirms the similar results. Notably, better survival rates and intervals were also found in mirtazapine-treated mice. These findings, however, were not observed in the immunodeficient mice. Our results suggest that tumor growth inhibition by mirtazapine in CT26/*luc* colon carcinoma-bearing mice may be due to the alteration of the tumor microenvironment, which involves the activation of the immune response and the recovery of serotonin level.

## Introduction

Antidepressant is prescribed for the treatment of patients with depression, and often for patients with advanced cancers as well [1]. A population-based nested case-control study reported that high dose of the selective serotonin reuptake inhibitor (SSRI), but not tricyclic antidepressant, before diagnosis decreased the risk of colorectal cancer by 30%, and suggested that anti-promoter effect or direct cytotoxic effect is possible [2]. Mirtazapine, a noradrenergic and specific serotonergic antidepressant (NaSSA), and certain SSRI antidepressants, such as fluoxetine, zimelidine, paroxetine, and sertraline, have been shown with antitumor effects in several human cancer cell lines [3]–[6]. However, a study with sertraline in patients with advanced cancers without major depression failed to show a beneficial effect on the survival [7]. Preclinical and clinical studies show that stress-related processes may impact pathways involved in cancer progression, invasion and immune-regulation [8]. Depletion in neurotransmitters, such as dopamine, under chronic stress may promote tumor growth by stimulating tumor microenvironment [9]. Furthermore, cytokine levels, especially interleukin-12 (IL-12), are reported to be affected by the depression [10], [11]. IL-12 stimulates T lymphocytes and natural killer cells to release interferon gamma (IFN-γ), which has been shown with the capability to inhibit tumor growth, angiogenesis, and metastasis both in rodents and human [12], [13].

**Figure 1:**
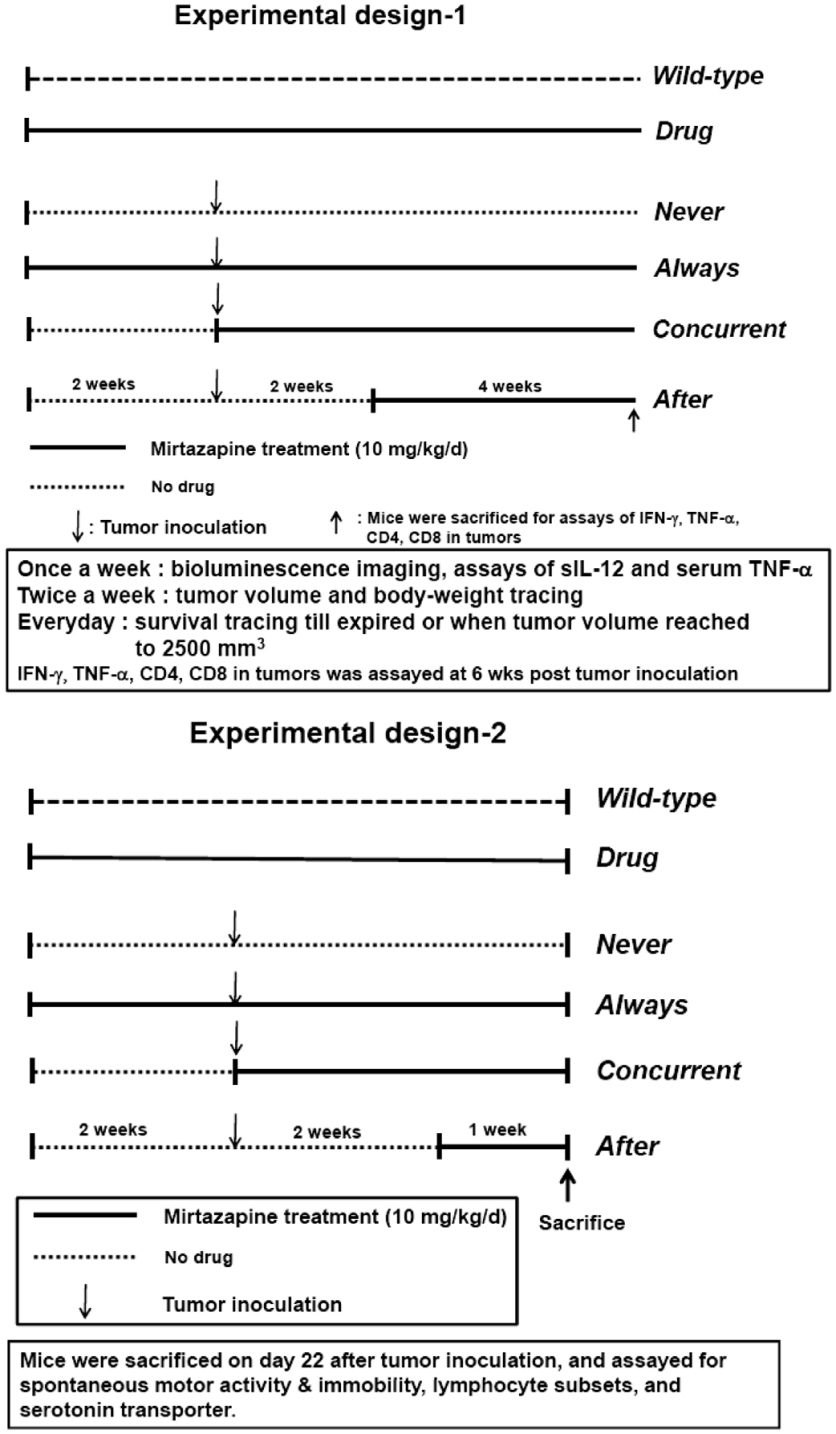
Experimental designs. (A) Tumor inoculation, mirtazapine treatment, and monitoring of tumor growth and survival. (B) On day 22, mice were assayed for behaviors, then sacrificed for the measurement of lymphocyte subsets and performed with *ex vivo* autoradiography.

Mirtazapine has been shown to be effective for mood disorder, insomnia, chemotherapy- and cancer-related nausea, poor appetite, and other distress symptoms in patients with cancers [14]–[18]. Mirtazapine is also an antagonist for the adrenergic alpha2-autoreceptors and alpha2-heteroreceptors with its high affinity for both 5-HT3 and 5-HT2A receptors [19], [20]. Clinical trial suggests that mirtazapine may be effective for improving multiple symptoms, including cachexia, anorexia, and quality of life in patients with advanced cancer [21], [22]. Whether mirtazapine is beneficial for the reduced risk of cancer incidence is worth to be investigated [23].

Here we established a CT26/*luc* colorectal carcinoma-bearing animal model combined with molecular imaging to investigate the effect of mirtazapine on tumor growth inhibition and its correlation with tumor microenvironment, such as immune-regulated factors and serotonin level, after the treatment with mirtazapine.

## Materials and Methods

### Tumor Cell Preparation

To evaluate the effect of mirtazapine on tumor growth inhibition, the CT-26 murine colon carcinoma cells (obtained from Taiwan Liposome Company, Taipei, Taiwan) were transfected with the luciferase gene (*luc*). The stable clone was maintained with 120 µg/ml G418 (Merck) as previously described in our study [45]. The CT26/*luc* tumor cells were cultured in RPMI 1640 medium (Invitrogen) supplemented with 10% fetal bovine serum (Hyclone), 100 units/ml of penicillin, and 100 µg/ml streptomycin (Gibco-BRL) at 37°C in a 5% CO_2_ atmosphere.

### Cell Viability and Cell Cycle Analysis

3-(4,5-Dimethylthiazol-2-yl)-2,5-diphenyltetrazolium bromide (MTT, Sigma, USA) was dissolved in phosphate-buffered saline (145 mM NaCl, 1.4 mM KH_2_PO_4_, 4.3 mM Na_2_HPO_4_, and 2.7 mM KCl, pH 7.2). CT26/*luc* cells were seeded in 96-well plates overnight, then treated with various concentrations (0, 5, 10, 20, 40, and 80 µM) of mirtazapine for 24, 48, and 72 h. Cell viability was determined with MTT assay. After washing with fresh medium, 100 µl of 1 mg/ml MTT solution was added to each well. After 4 hours incubation at 37°C, 100 µl DMSO was added to dissolve the MTT formazan, and the absorbance was determined with an ELISA reader (Power Wave X340, Bio-Tek Instrument Inc., USA) using a wavelength of 570 nm for the excitation.

CT26/*luc* cells were cultured in 10 cm-diameter dish (1×10^6^/dish) for 24 h, followed by the treatments with 0, 5, 10, 20, 40, and 80 µM mirtazapine (Megafine Pharma (P) Ltd., India). The cells were harvested in 15 ml centrifuge tubes 24 h later, fixed with cold 75% alcohol overnight. Cells were then centrifuged at 5000 rpm for 15 min at 4°C. After removal of the supernatant, cells were re-suspended in 0.8 ml cold phosphate-buffered saline (PBS), 0.1 ml RNase A (1 mg/ml; QIAGEN), and 0.1 ml propidium iodide (400 µg/ml) for 30 min at 37°C and kept in the dark to avoid quenching. The cell cycle analysis was assayed using a FACScan (BD Sciences) and analyzed by CellQuest software (BD Sciences).

**Figure 2:**
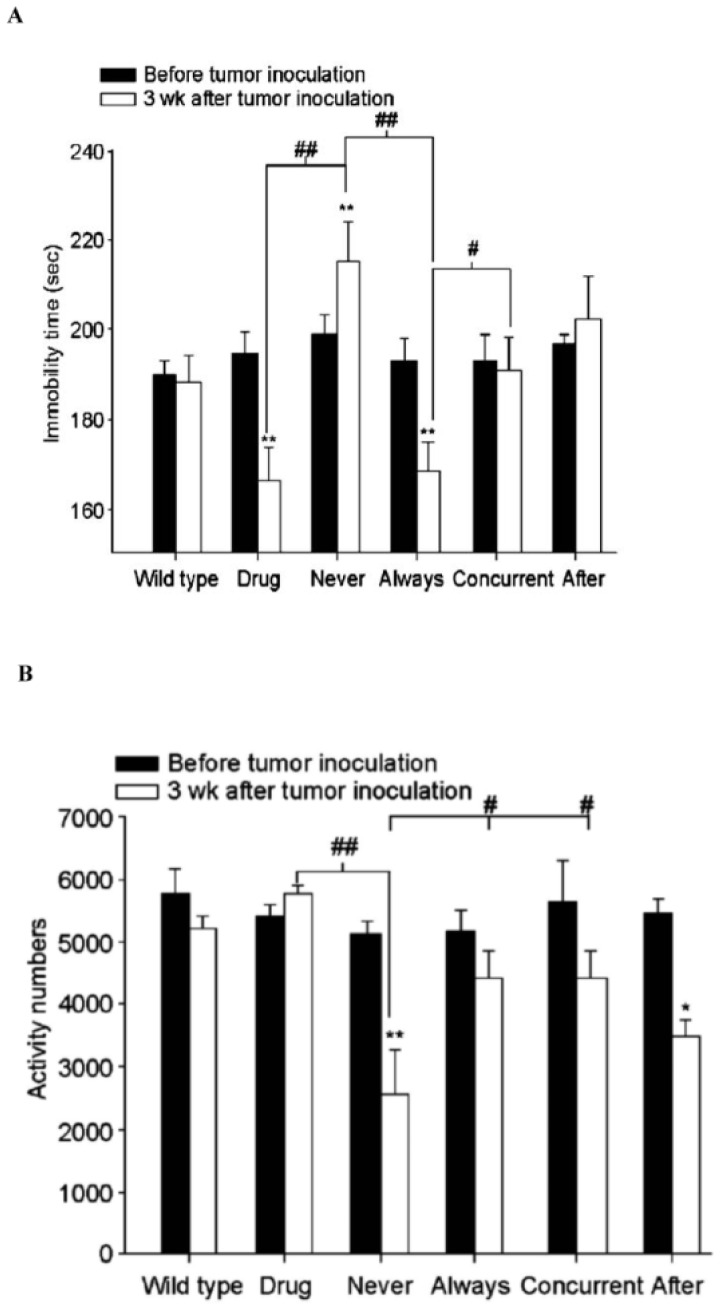
Effects of mirtazapine (10 mg/kg/d) on behavior changes of normal and CT26/*luc* tumor-bearing mice. (A) Immobility time in the tail-suspension test. (B) Spontaneous motor activity. (n = 4, *p<0.05, **p<0.01 vs. *wild-type*; ^#^ *p*<0.05, ^##^ *p* <0.01 between two groups).

### Tumor-bearing Animal Model

All animal study protocols were approved by the Institutional Animal Care and Use Committee (IACAU) of National Yang Ming University. Mirtazapine (0.25 mg) was dissolved in 0.05 ml of 0.9% NaCl plus 0.5% absolute ethanol for each mouse i.e. 10 mg/kg. Male BALB/c mice (initial weights 25±2 g) were housed in the cages, five mice per cage, under a 12∶12 h reverse light/dark cycle with lights off at 6 pm. Animals were handled and weighed daily for a week to reduce any non-specific stress responses. To study the effect of mirtazapine on the tumor growth inhibition, 6-weeks-old male BALB/c mice (25±2 gm, purchased from the National Laboratory Animal Center, Taipei, Taiwan) were randomly divided into 6 groups as shown in Figure 1A. (1) *wild-type*, no tumor inoculation and no mirtazapine treatment; (2) *drug*, continuous mirtazapine treatment without tumor inoculation; (3) *never*, tumor inoculation, no mirtazapine but daily 0.05 ml of 0.9% NaCl plus 0.5% absolute ethanol treatment; (4) *always*, mirtazapine treatment initiated 2 weeks before tumor inoculation; (5) *concurrent*, tumor inoculation and mirtazapine treatment on the same day; and (6) *after*, mirtazapine treatment initiated 2 weeks post tumor inoculation. The experimental design and the time for the biological end points were shown in Figure 1B.

**Figure 3:**
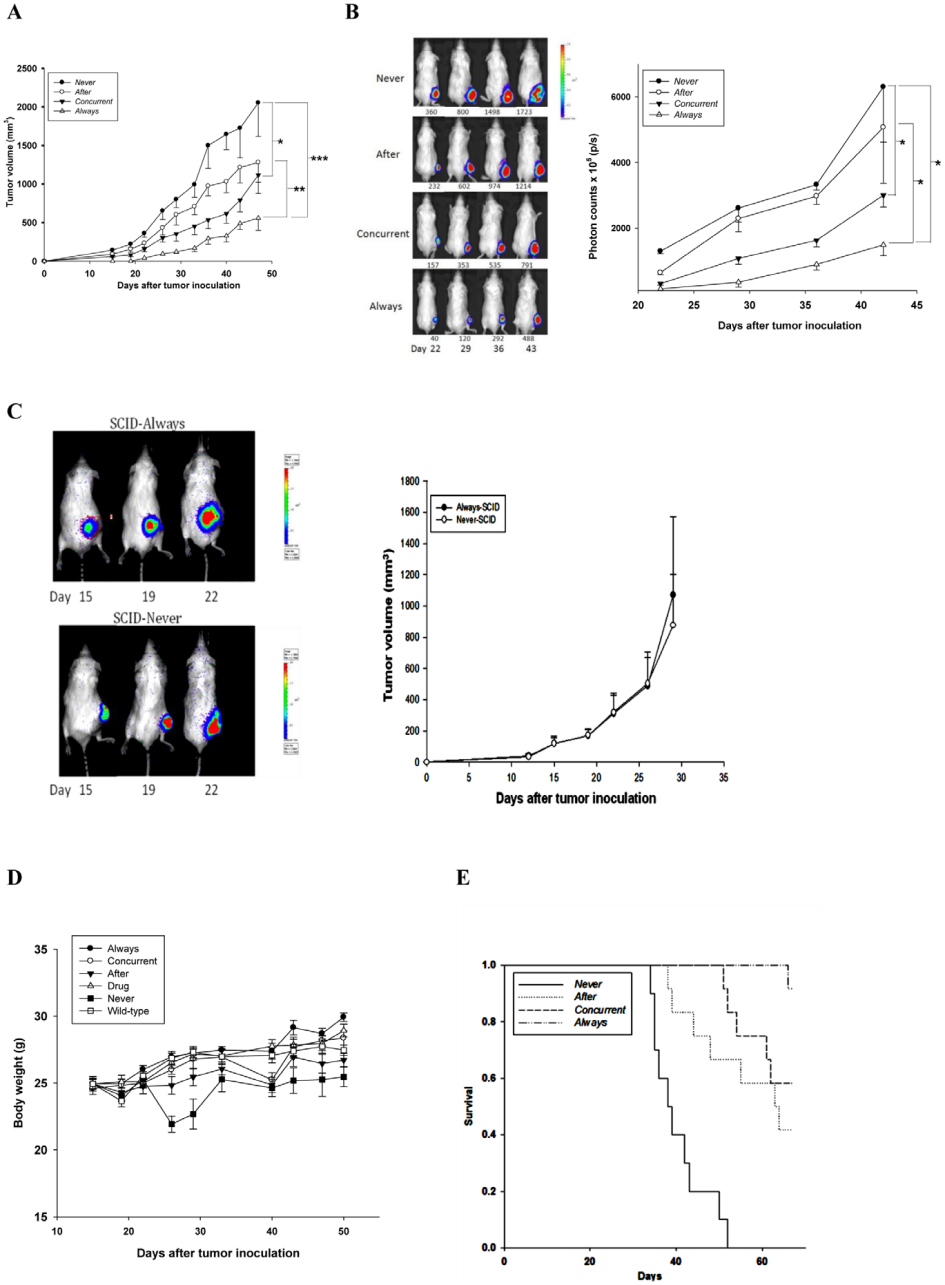
Mirtazapine inhibits tumor growth and prolongs the survival rate and interval in CT-26/*luc* tumor-bearing model. On day -14, only “Always” mice were inoculated with tumor cells and treated with mirtazapine throughout the experiment, the other three groups were treated with 0.05 ml of 0.9% NaCl plus 0.5% absolute ethanol up to day 0. On day 0, only “*concurrent*” mice were inoculated with tumor cells and treated with mirtazapine throughout the rest of the experiment, “*after*” mice were treated with 0.05 ml of 0.9% NaCl plus 0.5% absolute ethanol but without mirtazapine up to day 14, while “*never*” mice were treated with 0.05 ml of 0.9% NaCl plus 0.5% absolute ethanol and throughout the experiment. On day 14, “*after*” mice were inoculated with tumor cells and treated with mirtazapine throughout the rest of the experiment. (A) Tumor growth curves are monitored with digital caliper. (B) Left panel: tumor growth curves are monitored with noninvasive bioluminescence imaging (BLI). The value under each mouse is the tumor volume determined with a caliper. Right panel: quantification of the photon counts in ROIs from the left panel. (C) No antitumor effect of mirtazapine was found on immunodeficient SCID mice with CT26/*luc* tumors. Left panel: tumor growth curves for *always* and *never*. Right panel: quantification of the photon counts in ROIs from the left panel. (D) No significant body-weight change (within 20%) through the whole experiment was found among *wild-type*, *drug*, *never*, *always*, *concurrent*, and *after*. (E) Effects of mirtazapine on the survival rate and interval of CT26/*luc*-bearing mice. The mean survival times are 67, 64, 57, 43 days for *always*, *concurrent*, *after*, and *never*, respectively. (n = 10 per group, *p<0.05, **p<0.01, ***p<0.001).

**Table 1:** Tumor growth inhibition among *never, after, concurrent*, and *always* of mice.

Group	n	Mean tumorgrowthtime^a^ (day)	Mean tumor growthdelay time^b^ (day)	Mean growthinhibition rate^c^
*never*	12	22.5	NA^d^	NA
*always*	12	41.3	18.8	1.8
*concurrent*	12	30.9	8.3	1.4
*after*	12	25.4	2.8	1.1

a Mean tumor growth time: the time at which the tumor volume reaches to 400 mm^3^.

b Mean tumor growth delay time: the tumor growth time of the treated group minus that of the *Never*.

c Mean growth inhibition rate: growth rate of treated group/ growth rate of *Never*.

d NA: not available.

CT26/*luc* cells (2×10^6^ cells/200 µL) suspended in the serum-free RPMI medium were transplanted subcutaneously into the dorsal region of the right thighs of the BALB/c mice (purchased from the National Laboratory Animal Center, Taiwan). 10 mg/kg/d mirtazapine [46], [47] dissolved in 0.9% sodium chloride and 0.5% ethanol was administered to mice by gavage daily till mice expired or terminated on day 67 post tumor inoculation. Survival rate and interval were assayed for *never*, *always*, *concurrent*, and *after* (n = 10 per group).

Six-weeks-old immunodeficient male SCID mice (purchased from the National Laboratory Animal Center, Taiwan) were also used to verify the involvement of the immune system in the inhibition of the tumor growth by mirtazapine. The SCID mice were divided into 2 groups: (1) *never-SCID,* tumor inoculation but no mirtazapine treatment, and (2) *always-SCID,* mirtazapine treatment initiated 2 weeks before tumor inoculation.

### Tumor Volume and Mice Activity Assays

Tumor growth was monitored using a digital caliper twice a week. The tumor volume was calculated according the formula: 0.523× length × width × thickness. Bioluminescence imaging (BLI) used for tumor size tracking was performed with an IVIS50 animal imaging system (Xenogen Corp., USA) as previously described [48].

The behavioral change in the animal depression model was evaluated as previously described [49]. For the spontaneous motor activity assay, the mouse was placed in a separate chamber and allowed to rest for 3 min. The number of movements was automatically counted during a 5-min period (Process Control, ActiMot 302020, TSE Systems). On the other hand, the duration of immobility was assayed with the tail suspension test [50]. Acoustically and visually isolated mouse was suspended at the tip of the tail with 50 cm high above the floor. Immobility time was recorded for 6 min.

**Figure 4:**
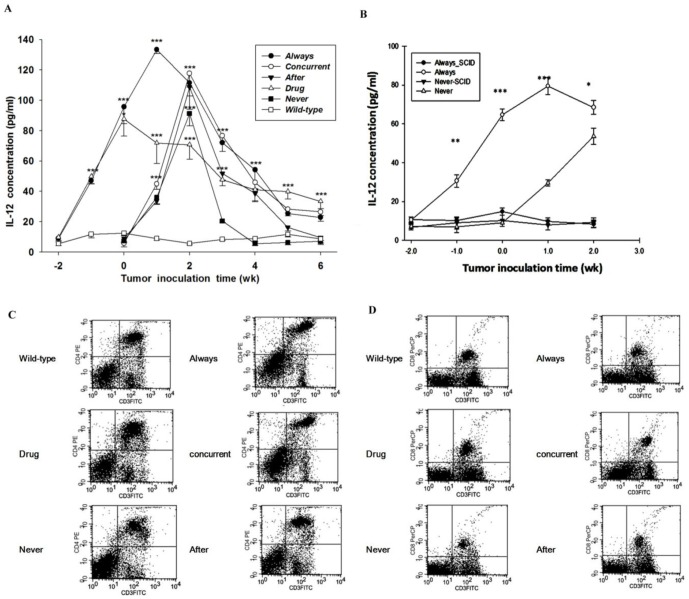
Immunocompetence analysis in CT26/*luc*-bearing mice. (A) The sIL-12 levels vs. time before and after tumor cell inoculation. The peak sIL-12 levels were found at 1 and 2 days post tumor cell inoculation for *always* and the rest groups, respectively, except *drug*, which was at day 0. (***p<0.001 vs. *wild-type*) (B) The sIL-12 levels remain low and unchanged in *always*-SCID and *never*-SCID mice as a function of time before and after tumor inoculation. (n = 10, *p<0.05, **p<0.01, and ***p<0.001 vs. *never*) (C) CD4 PE vs. CD3 FITC T lymphocytes determined with flow cytometer. (D) CD8-PerCP vs. CD3 FITC T lymphocytes determined with flow cytometer. Both (C) and (D) are also tabulated in Table 2.

### Immunocompetence Evaluation and Immunohistochemistry of Serum Interleukin-12, CD4+ and CD8+ in the Blood, Lymph Nodes and Tumors

For quantification of IL-12, the whole blood withdrawn from the pouch of each mouse was centrifuged at 600×*g* for 20 min, and serum was collected. The serum IL-12p70 (sIL-12) level was determined using an ELISA kit (R&D Systems, Taiwan). Identification for the lymph node cluster of differentiated CD4+ T helper and CD8+ T-cytotoxic lymphocyte subsets was assayed [27]. Briefly, the lymphocytes isolated from the lymph nodes of groins of mice were stained with phycoerythrin-conjugated anti-mouse CD4 (CD4-PE) monoclonal antibody and peridinin-chlorophyll-protein-complex-conjugated anti-mouse CD8 (CD8-PerCP) monoclonal antibody (BioLegend, USA). Lymphocyte subsets were identified by FACS analysis using a FACS Calibur flow cytometer (BD Sciences, USA). Immunohistochemistry (IHC) of CD4 and CD8 was also performed on day 42 post tumor inoculation. Tumors were removed, paraffin embedded, and 5-µm sectioning was performed. The sections were immunohistostained with antibodies against CD4 (BioLegend, USA) and CD8 (BioLegend, USA), respectively. The procedures of immunohistostaining were followed the protocols provided with the IHC kit (Millipore, USA). All images were digitally captured on a Scanscope CS system (Aperio, USA).

The level of IFN-γ in the tumor was determined using an ELISA kit (R&D Systems, Taiwan). Briefly, 6 weeks after tumor inoculation, the mice were sacrificed and the tumors were quickly removed and minced, then added with lysis buffer containing 1% protease inhibitor cocktail (T-PER tissue protein extraction reagent, Thermo Scientific, USA). After sonication, the cell mixture was centrifuged with 15000 rpm (Kubota centrifuge 1700, Japan) at 4°C for 10 min. The supernatant was collected for the protein quantification using bovine serum albumin as the standard. Two mg of the tumor proteins was used for the quantification of IFN-γ.

**Table 2:** The CD4+ and CD8+ T cell subsets with or without mirtazapine treatments in BALB/c mice with or without CT26/*luc* tumors.

Group	CD4+ T cells(10^4^ events)	CD8+ T cells(10^4^ events)
*Wild type*	32.63±1.36%	28.80±7.00%
*Drug*	30.97±1.40%	30.95±6.57%
*Never*	17.49±1.07%^***^	12.76±3.10%*
*Always*	29.75±1.96%^###, +^	32.77±7.43%^#^
*Concurrent*	25.77±0.73%^###, +^	22.41±5.03%
*After*	22.58±1.15%^##^	15.86±4.78%

n = 6, ^*^ *p*<0.05, ^**^ *p*<0.01, and ^***^ *p*<0.001 as compared with that of *wild type*, ^#^ *p*<0.05, ^##^ *p*<0.01, and ^###^ *p*<0.001 as compared with that of *never*, ^+^ *p*<0.05 as compared with that of *after*.

**Table 3:** Effect of mirtazapine on IFN-γ levels in tumors* of CT-26/*luc* tumor-bearing mice.

Group	IFN-γ (pg/ml)
*Never*	4.10±0.25
*Always*	85.35±4.50^##, ++^
*Concurrent*	39.42±7.42^#, +^
*After*	19.60±1.13^#^

n = 3/group, ^##^ *p*<0.01 and ^#^ *p*<0.05 as compared with that of *never*, ^++^ *p<*0.01 and ^+^ *p<*0.05 as compared with that of *after*. Student’s *t* test was used for the analysis. Experiments were repeated twice.

* The tumors were removed from the mice at 6 weeks post tumor cells inoculation.

### Effects of Mirtazapine on TNF-α expressions in the Blood Circulation and Tumor Tissues

For quantification of TNF-α, the whole blood withdrawn from the pouch of each mouse once a week for up to 6 weeks was centrifuged at 600×*g* for 20 min, and serum was collected. The serum TNF-α level was evaluated with an ELISA kit (eBioscience, USA). The level of TNF-α in the tumor of mice on day 42 post tumor inoculation was determined using ex vivo Western Blotting assay. Briefly, 6 weeks after tumor inoculation, the mice were sacrificed and the tumors were quickly removed and minced, then added with lysis buffer containing 1% protease inhibitor cocktail (T-PER tissue protein extraction reagent, Thermo Scientific, USA). Equal amounts of proteins (40 µg) were subjected to SDS-PAGE and transferred to PVDF membranes (Millipore, Bedford, MA). Non-specific binding was blocked by incubation with 5% non-fat milk. Membrane was incubated with antibodies against TNF-α (Abbiotec, USA) and β-actin (Millipore, USA) overnight at 4°C. The goat-anti rabbit IgG (Millipore) and goat-anti mouse IgG conjugated with horseradish peroxidase (Millipore) were used as the secondary antibodies. The band signal from the antigen-antibody binding was illustrated with enhanced chemoluminescence system (ECL, Millipore). Image J software (National Institutes of Health, USA) was used for the quantitative analysis.

### Uptake of [^123^I]ADAM in the Brain with Quantitative Autoradiography

The uptake of 2-((2-((dimethylamino)methyl)phenyl)thio)-5-iodophenylamine ([^123^I]ADAM) in the moue brain was assayed as previously described [42]. CT26/*luc* tumor-bearing mice were injected with 1 mCi/0.1 ml of [^123^I]ADAM (purchased from the Institute of Nuclear Energy Research, Taiwan) via the caudal vein, and sacrificed at 90 min post injection, and assayed with ex vivo autoradiography. Briefly, the brain slices (5 µm thickness) were put onto an imaging plate (BAS cassette 2340, FujiFilm, Japan), and exposed for 24 hours. The imaging plates were then scanned with a high-resolution imaging plate reader (FLA5000, FujiFilm, Japan) at the following settings: resolution 25, gradation 16 bits, and dynamic range L5. The specific binding ratio (SBR) was calculated as the following: SBR  =  (target – cortex)/cortex.

**Figure 5:**
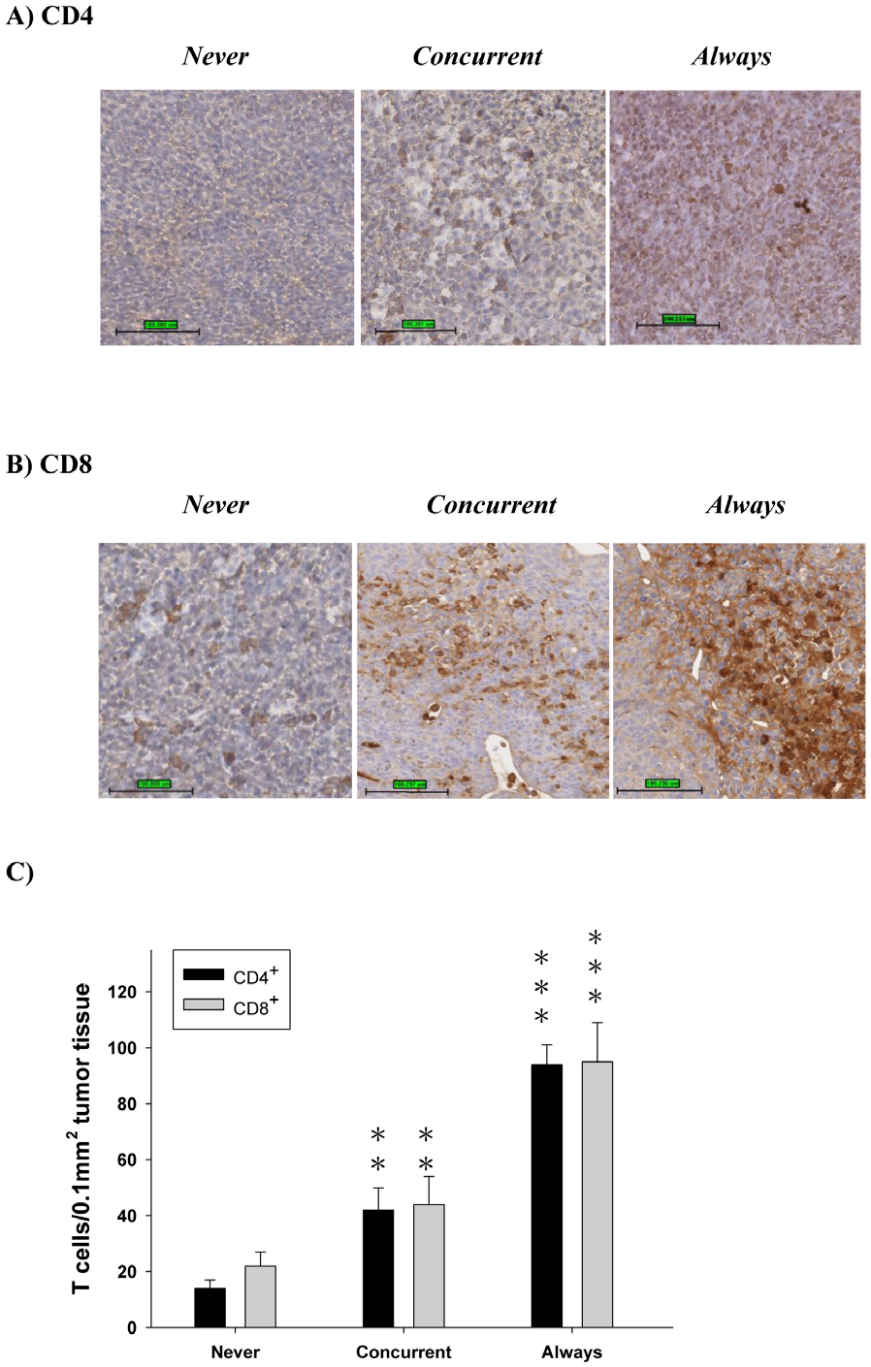
Immunohistostaining of infiltrating CD4+ and CD8+ T cells in tumor tissues of mirtazapine-treated, i.e. *concurrent* and *always*, and *never* mice. (A) CD4 and (B) CD8 in tumors were performed on day 42 post tumor inoculation. Magnification 200 ×. (C) Quantification of CD4+ and CD8+ T cells. (n = 3, **p<0.01 and ***p<0.001 as compared to those of *never*).

### Statistical Analysis

All data were shown as the mean±standard error. Student’s *t* test was used for the comparison between two groups. Kaplan-Meier plotting was used for the survival analysis, and was compared using the log-rank test. Differences between the means were considered significant if *p*<0.05 or less.

## Results

### Luciferase Gene Expression and Cytotoxicity of Mirtazapine on CT26/luc Cells

Both CT-26 and CT-26/*luc* cells show the similar growth curves with doubling time of 14 hrs. Three photons/cell/sec of CT-26/*luc* cell line were found with luciferase gene expression assay. No cytotoxicity was found in the CT26/*luc* cells treated with 5–80 µM mirtazapine for 24, 48, and 72 hrs (Figure S1). Flow cytometric analysis also shows the similar result. These results are shown in the supplement.

### Animal Behavior

The spontaneous motor activity and immobility time of mice were evaluated on day 22 after tumor inoculation and with or without mirtazapine intervention. The increase in the immobility time and the decrease in the number of spontaneous motor activity were observed after the implantation of CT26/*luc* tumors as shown with *never*. Continuous administration of mirtazapine significantly decreased the immobility time, but had no effect on the spontaneous motor activity as shown with *drug* and *always* (Figures 2A and 2B).

**Figure 6:**
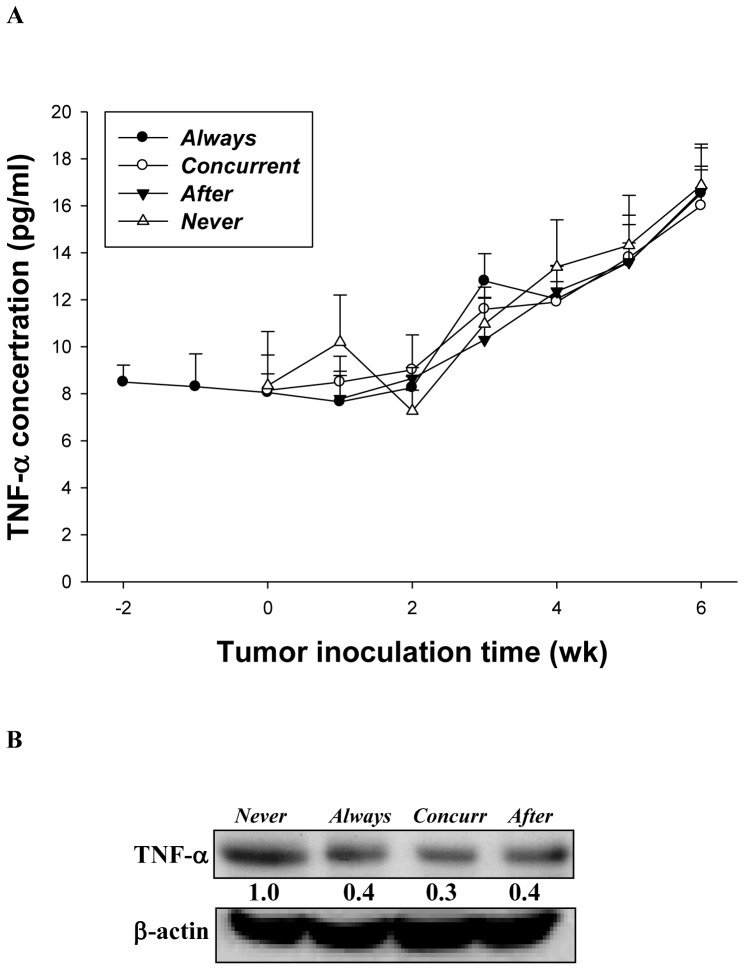
Effects of mirtazapine on TNF-α expressions in the blood circulation and tumors, respectively. (A) The serum TNF-α level was evaluated with ELISA once a week for up to 6 weeks post tumor inoculation, n = 10 for each group; (B) The TNF-α levels were assayed with *ex vivo* Western blotting in tumor tissues of mice on day 42 post tumor inoculation.

### Mirtazapine Suppresses Tumor Growth and Improves Survival of Tumor-bearing Mice

Significant tumor growth inhibition (*p*<0.01) was found in all mirtazapine-treated groups (*always, concurrent, after*) as compared to that of the *never* from day 22–47 after tumor inoculation. Tumor growth delay of the *always* was significant higher than those of the *concurrent* and *after* groups (*p*<0.01) as shown in Figure 3A. BLI also confirmed the similar results (Figure 3B). The tumor inhibition effect of mirtazapine, however, was not found in SCID mice as shown in Figure 3C. In addition, no significant body weight change throughout the experiment was found among all groups indicated no general toxicity with mitazapine treatment (Figure 3D). The overall survival times (Figure 3E) for mirtazapine-treated, tumor-bearing mice (*always, concurrent, after*) were all significantly longer than that of the *never* (43.1±2.6 days). The survival times for *always*, *concurrent*, and *after* were 66.9±0.1, 63.6±1.5, and 57.0±3.2 days, respectively. The survival time of *always* was significantly longer than that of the *concurrent* (*p*<0.01). Table 1 shows the mean tumor growth inhibition rates of *never, after, concurrent*, and *always*, respectively. Mice treated with mirtazapine two weeks prior to the tumor inoculation (*always*) showed the highest inhibition of tumor growth.

**Figure 7:**
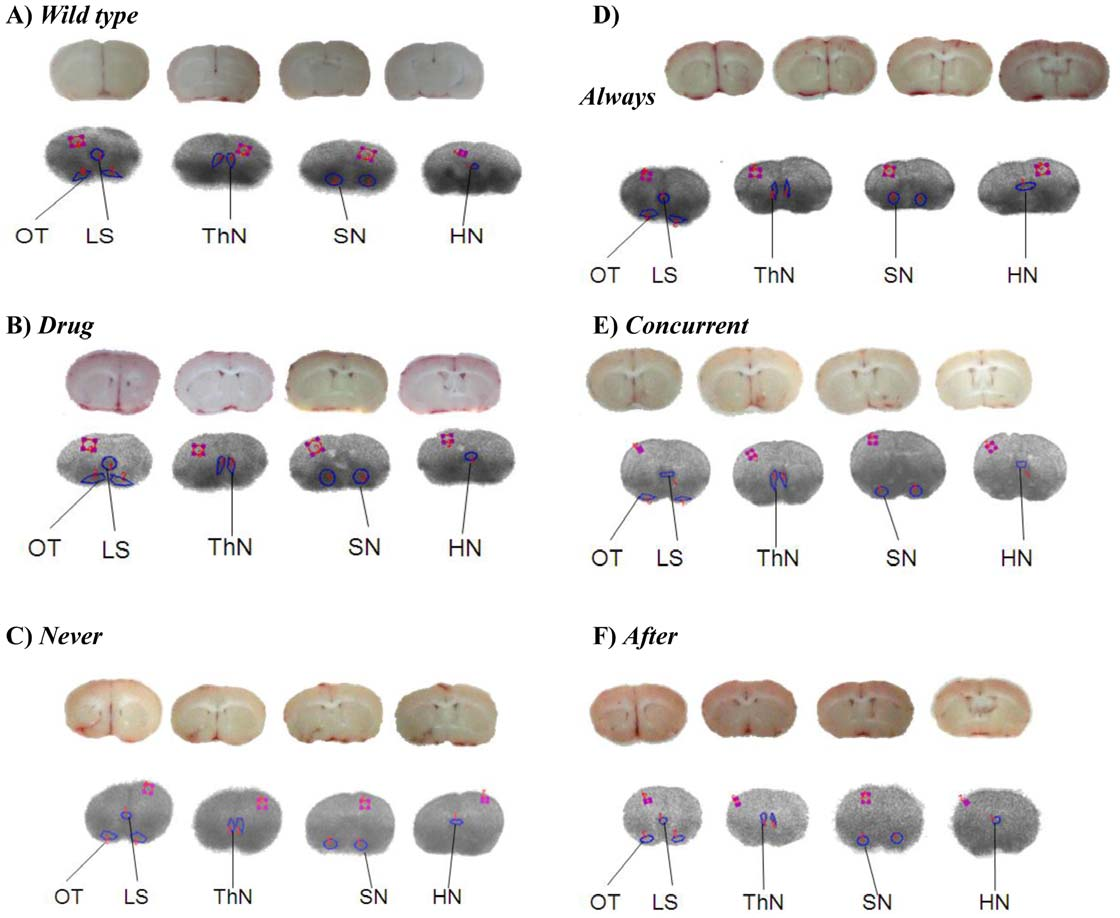
Serotonin transporter determined with [^123^I]ADAM/*ex vivo* autoradiography in the brain of CT26/*luc* tumor-bearing mice. The mouse brain obtained at 90 min post caudal vein injection of 1 mCi/ml [^123^I]ADAM was performed with *ex vivo* autoradiography. The top and bottom rows in each subfigure are the anatomy and *ex vivo* autoradiography, respectively. The blue circle is the target, and the red square is the cortex. (OT  =  olfactory tubercle; LS  =  lateral septal nucleus; ThN  =  thalamic nuclei; SN  =  substantia nigra; HN  =  hypothalamic nuclei).

### Mirtazapine Alters Cytokine Production and Increases CD4+/CD8+ T Cell Counts

Figure 4A shows that sIL-12 concentrations are increased to the peak levels with 13 and 18 folds at 0 and 1 wk post tumor cell inoculations for *drug* and *always,* respectively. On the other hand, sIL-12 concentrations were increased 17, 16 and 13 folds for *concurrent, after and never*, respectively. Notably, the sIL-12 concentration of *never* returns to the normal level, but *drug* still remains high (42 vs. 7 pg/ml) at 4 wks post tumor cell inoculation. The results suggest that the effect of tumor growth on sIL-12 level is less than that of continuous mirtazapine treatment, especially when drug administration is prior to tumor inoculation. The sIL-12 concentrations of *always* and *concurrent* were still significantly higher than that of *after*, the latter dropped to the control level at 6 weeks post tumor inoculation (*p*<0.01 and *p*<0.05, respectively). The increase of sIL-12 level after mirtazapine treatment, however, was not found in the SCID mice as shown in Figure 4B. In addition, both CD4+ and CD8+ T cell counts were lower in CT26/*luc* tumor-bearing mice (*never*), but not in the mirtazapine-treated, tumor-bearing mice (*always*, *concurren*t, and *after*) as compared with those of *wild type* and *drug* (Table 2). Both CD4+ and CD8+ T cell counts of *always* were the highest among the three mirtazapine-treated, tumor-bearing animal groups (Figures 4C and 4D). The expression of IFN-γ in tumors was significantly higher in *always, concurrent*, and *after* as compared with that of *never*, with the highest expression in *always* (Table 3). In addition, earlier mirtazapine intervention, such as *always* and *concurrent*, resulted in significantly higher IFN-γ expression as compared with that of *after*. Notably, Figures 5A and 5B show that significantly increased numbers of infiltrating CD4+ and CD8+ cells/0.1 mm^2^ tumor tissues of “*concurrent*” and “*always*” as compared with those of “*never*”, and were quantified in Figure 5C, *p<*0.01 and *p*<0.001, respectively.

**Table 4:** Specific binding ratios of [^123^I]ADAM in brains of BALB/c mice with or without CT26/*luc* tumors determined with *ex vivo* autoradiography.

Group	Specific binding ratio				
	LS	OT	ThN	SN	HN
*Wild-type*	1.45±0.05	1.36±0.10	1.23±0.14	2.58±0.10	1.55±0.12
*Drug*	1.77±0.10*,#	1.95±0.16*,##	1.72± 0.09*,##	2.81±0.08^**,###^	2.14±0.12*,##
*Never*	1.13±0.07*	1.12± 0.06*	0.93± 0.15*	1.47± 0.07*	1.28± 0.12*
*Always*	2.00±0.04^**,###,<$>\scale30%\raster="rg2"<$><$>\scale30%\raster="rg2"<$><$>\scale30%\raster="rg2"<$>^	2.01±0.08^**,###,<$>\scale30%\raster="rg2"<$>^	1.99± 0.07^**,###,<$>\scale30%\raster="rg2"<$><$>\scale30%\raster="rg2"<$>^	2.46±0.07^###^	2.29± 0.10^**,##^
*Concurrent*	1.78±0.05^**,##,<$>\scale30%\raster="rg2"<$><$>\scale30%\raster="rg2"<$>^	1.91±0.09*,##	1.76±0.07^**,###,<$>\scale30%\raster="rg2"<$>^	2.44±0.06^###^	2.227± 0.132*,##
*After*	1.36± 0.03^#^	1.63±0.12^#^	1.47±0.06^##^	2.17±0.16^#^	2.11± 0.12*,##

*Ex vivo* autoradiography was performed at 90 mins post i.v. injection of 1 mCi [^123^I]ADAM/0.1 ml. Specific binding ratio  =  (target – cortex)/cortex. (OT  =  olfactory tubercle; LS  =  lateral septal nucleus; ThN  =  thalamic nuclei; SN  =  substantia nigra; HN  =  hypothalamic nuclei).

* *p*<0.05, ^**^ *p*<0.01 vs. *wild-type*; **^#^** *p*<0.05, **^##^** *p*<0.01, **^###^** *p*<0.001 vs. *never*, **^<$>\scale30%\raster="rg2"<$>^** *p*<0.05, **^<$>\scale30%\raster="rg2"<$><$>\scale30%\raster="rg2"<$>^** *p*<0.01, **^<$>\scale30%\raster="rg2"<$><$>\scale30%\raster="rg2"<$><$>\scale30%\raster="rg2"<$>^** *p*<0.001 vs. *after*. Data are means±S.E. n = 3/group. Experiments were repeated twice.

### Effects of Mirtazapine on TNF-α Expressions in the Blood Circulation and Tumor Tissues

The serum TNF-α level was evaluated with enzyme-linked immunosorbent assay (ELISA) once a week for up to 6 weeks post tumor inoculation. Figure 6A shows that the serum TNF-α levels are gradually increased from the third weeks up to six weeks post tumor inoculation, however, no significant difference is found among tumor-bearing mice treated with and without mirtazapine, respectively. On the other hand, the TNF-α levels in tumors of mice (*Always*, *Concurrent*, and *After*) assayed with *ex vivo* Western blotting on day 42 post tumor inoculation were decreased to 40% of that of “*Never*” as shown in Figure 6B.

### Mirtazapine Enhances Serotonin Levels in the Brains of Tumor-bearing Mice

The higher uptake of [^123^I]ADAM by serotonin transporter (SERT)-rich areas, such as olfactory tubercle, lateral septal nucleus, thalamic nuclei, substantia nigra, and hypothalamic nuclei, in the brain is shown in Figure 7 as determined with *ex vivo* autoradiography. The specific binding ratios (SBRs) of [^123^I]ADAM in SERT-rich areas of mice are listed in Table 4, in which specific binding ratio  =  (target – cortex)/cortex. SBRs were significantly higher in *drug* as compared with those of *wild type* (*p*<0.05). SBRs in *always*, *concurrent*, and *after* were also significantly higher than those of *never* (*p*<0.05). The results are in accordance with that SERT-rich areas are more susceptible to mirtazapine treatment. In addition, earlier mirtazapine intervention, as *always* and *concurrent*, contributes to a more significant increase of SBRs as compared with that of *after* (*p*<0.01).

## Discussion

The tail suspension test has been reported as a well established method for the activity of antidepressants, and the spontaneous motility is a useful measure of overall behavior of the mice [24]. In this study, mice treated with and without mirtazapine, respectively, showed that “*Drug*” (no tumor), and “*Always*” (with tumor), were shortest in the immobility time among all groups at 3 weeks post tumor inoculation. The result suggests that mirtazapine may resolve the anxiety and depression in tumor-bearing mice as those found in cancer patients [15], [17].

Some SSRIs and tricyclic antidepressants contribute to the successful antidepressant therapy mainly through decreasing the production of pro-inflammatory cytokines, such as IFN-γ, and increasing the anti-inflammatory cytokines [25]–[27]. Nevertheless, it remains unclear whether immune response plays a causative role in the pathophysiology of depressive disorders. The increased sIL-12 levels in patients with major depressive disorders have been reported to be decreased after the treatment with antidepressants, including nefazodone, paroxetine, fluoxetine, sertraline, and venlafaxine [10], [26]. sIL-12, a multifunctional cytokine, is recognized as a key regulator for the cell-mediated immune responses [12], [13], [28], [29]. Preclinical trials show that the immunomodulatory and anti-angiogenic functions of sIL-12 are through the activation of innate cells (NK and NK-T cells) and adaptive immune response (CD4+ and CD8+ T cells), priming the secretion of IFN-γ [28]. The antitumor effect of sIL-12 in patients treated with continuous administration of antidepressants, however, is gradually reduced and limits its clinical application [28], [30], [31]. On the other hand, the IFN-γ levels in the whole bloods obtained from healthy volunteers were inhibited when treated with antidepressants [20], [32], [33].

Here we found that *in vivo* chronic mirtazapine treatment could inhibit the tumor growth and prolong the survival of tumor-bearing mice, which showed increased serum IL-12 level, CD4+, CD8+ in the lymph nodes, as well as serotonin transporters in the brain, and decreased TNF-α and IFN-γ in the tumors. The increased sIL-12 levels in mirtazapine-treated mice are maintained above the pre-therapy levels for more than four weeks, especially those with early mirtazapine intervention, such as *always* which show the highest survival rate and time with the highest increase of sIL-12 levels and the uptake of [^123^I]ADAM, a radiophamaceutical for serotonin transporter. Immunodeficient mice, on the other hand, do not show the similar effects when treated with mirtazapine. Both CD4+ and CD8+ T cells, may also contribute to the anticancer effect since their counts are recovered in those tumor-bearing mice treated with mirtazapine (Table 2).

The IFN-γ levels in tumors of mice treated with mirtazapine are significantly higher than those untreated, suggest that the immune response may be also involved in the antitumor effect of mirtazapine similar as the finding reported by Frick et al. [34]. Although certain antidepressants show pro-apoptotic effect on human colon carcinoma cell lines [4], [6], [35], mirtazapine is non-toxic to CT26 colon carcinoma as shown in this study. The plasma levels of TNF-α and soluble TNF receptors are increased in patients with major depressive disorders treated with mirtazapine [36]. With norepinephrine transporter knockout mice, Kubera et al. found that the decrease of IL-6 and IFN-γ, and the increase of IL-4 production may be due to the increase of norepinephrine level in the spleen after mirtazapine treatment [37]. On the other hand, IFN-γ-indoleamine 2,3-dioxygenase (IDO) axis also has been reported to regulate the sIL-12-mediated antitumor immunity [28], in which IFN-γ is the main cytokine induced by sIL-12 and plays a critical role to its antitumor effects [38]. IDO is highly inducible by pro-inflammatory cytokines, including IFN-γ and tumor necrosis factor-α (TNF-α). IDO is the first and rate-limiting enzyme involved in the tryptophan-kynurenine pathway. Degradation of tryptophan through the kynurenine pathway shows important neuropsychiatric implications. In addition, IDO is expressed in the brain so that fluctuations in its enzymatic activity can affect serotonin biosynthesis [39]. Decreased tryptophan concentration affects the serotonergic neurotransmission in the brain. Therefore, adequate physiological serotonin levels are indispensable for cytokine production. Mirtazapine may have a role in restoration of the equilibrium between physiological and pathological levels of cytokines in the brain [26], [40]. Whether IDO is involved in the immune response and serotonin recovery in cancer patients treated with mirtazapine is worth to be further studied.

In our previous study, we have reported that [^123^I]ADAM is an useful radiophamaceutical for diagnosing serotonin transporter (SERT) location sites in central nervous system (CNS), peripheral nervous system (PNS), and neuroendocrine tissues/organs, such as mucosa of the stomach and medulla of the adrenal glands [41]. The SERT-rich regions in the mouse brain can also be determined with ex vivo autoradiography using [^123^I]ADAM [42]. Although only the higher specific SERT binding sites in the midbrain for [^123^I]ADAM with *ex vivo* autoradiography were shown in this study (Figure 7), the PNS and neuroendocrine tissues/organs should have the higher uptake of [^123^I]ADAM as well. SERT availability in the midbrain of healthy subjects imaged with [^123^I]ADAM/SPECT has been shown to correlate with the overall rating scores and the life quality [43]. Here, we found that the lower uptake of [^123^I]ADAM in the midbrain of tumor-bearing mice could be recovered when treated with mirtazapine. Since the quality of life can be used as a prognostic factor in cancer patients [44], its improvement by mirtazapine may also contribute to the overall survival via normal serotonergic activity in the brain of subject.

A study performed by Xu et al. shows that selective serotonin reuptake inhibitors (SSRI) may reduce the risk of human colorectal cancer [2]. Our result shows that the most therapeutic efficacy for cancer treatment is “*Always*”, where the mice are pretreated with mirtazapine, a tetracyclic antidepressant, for two weeks before tumor cell injection. This finding implies that mirtazapine may also exert the similar therapeutic effect on tumor prevention as do those selective serotonin reuptake inhibitors (SSRI). This might also be interpreted as an effect on tumor establishment/prevention, or perhaps that the mirtazapine needs several weeks to take effect if it is an indirect effect on the serotonin and then the cytokines.

In conclusion, the better tumor growth inhibition and the longer survival rate and time are found in tumor-bearing mice treated with mirtazapine, especially in those with early intervention. Our results suggest that the antitumor effect of mirtazapine in CT26/*luc* colon carcinoma-bearing mice is via the activation of the immune response and the recovery of serotonin level in serotonergic system.

## Supporting Information

Figure S1 (A) The growth curves of parental CT-26 and CT-26/*luc* tumor cells. Td = (t-t_0_)×ln2/ (lnN – lnN_0_). The cell doubling times are 14.2 h and 14.4 h for parental CT-26 and CT26/*luc* cells, respectively. (B) Left: the luciferase expression in CT-26/*luc* cells imaged with Xenogen IVIS 50 imaging system. Right: the photon counts emitted from CT-26/*luc* cells is the function of the cell number with R^2^ = 0.993. (C) Cell viability analysis of CT26/*luc* cells treated with various concentrations (0, 5, 10, 20, 40, and 80 µM) of mirtazapine for 24, 48, and 72 h, and measured with MTT assay as described in the “Materials and Methods”. (D) Effect of mirtazapine on the cell cycle of CT-26/*luc* cells after treatment with various concentrations of mirtazapine for 24 h, and analyzed by flow cytometry. No cytotoxic effect was found.
(DOC)
