# Supplementary material for: Mirtazapine Inhibits Tumor Growth via Immune Response and Serotonergic System
Source: PLoS One. 2012 Jul 13;7(7):e38886. doi: 10.1371/journal.pone.0038886 (PMC3396612; doi:10.1371/journal.pone.0038886)

**Supplemental results**

**Fig. 1** (A) The growth curves of parental CT-26 and CT-26/*luc* tumor cells. Td = (t-t_0_) × ln2/ (lnN – lnN_0_). The cell doubling times are 14.2 h and 14.4 h for parental CT-26 and CT26/*luc* cells, respectively. (B) Left: the luciferase expression in CT-26/*luc* cells imaged with Xenogen IVIS 50 imaging system. Right: the photon counts emitted from CT-26/*luc* cells is the function of the cell number with R^2^ = 0.993. (C) Cell viability analysis of CT26/*luc* cells treated with various concentrations (0, 5, 10, 20, 40, and 80 μM) of mirtazapine for 24, 48, and 72 h, and measured with MTT assay as described in the “Materials and methods”. (D) Effect of mirtazapine on the cell cycle of CT-26/*luc* cells after treatment with various concentrations of mirtazapine for 24 h, and analyzed by flow cytometry. No cytotoxic effect was found.

1A


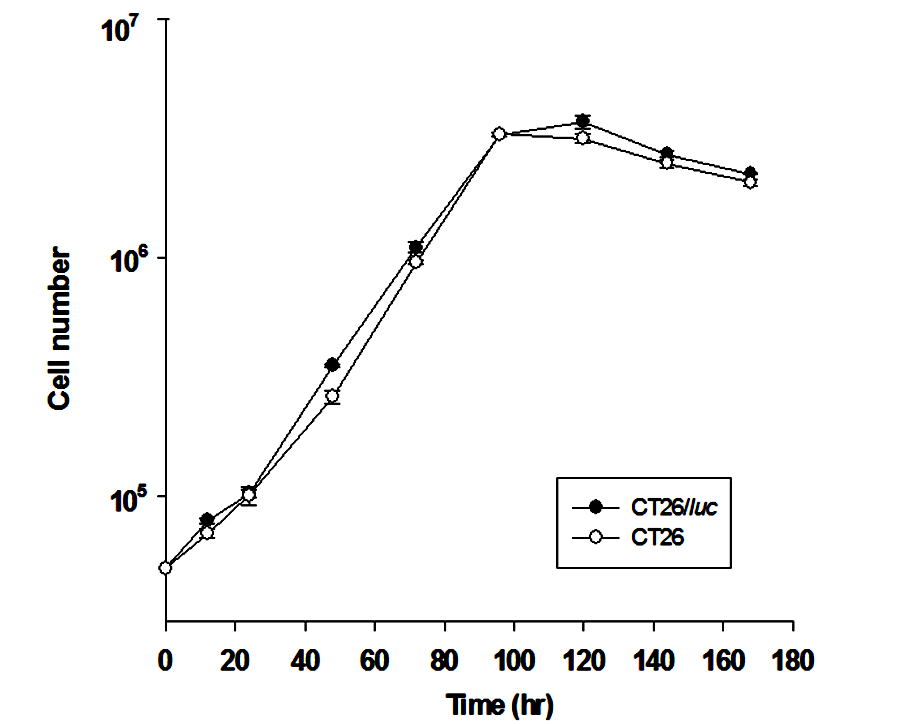


1B


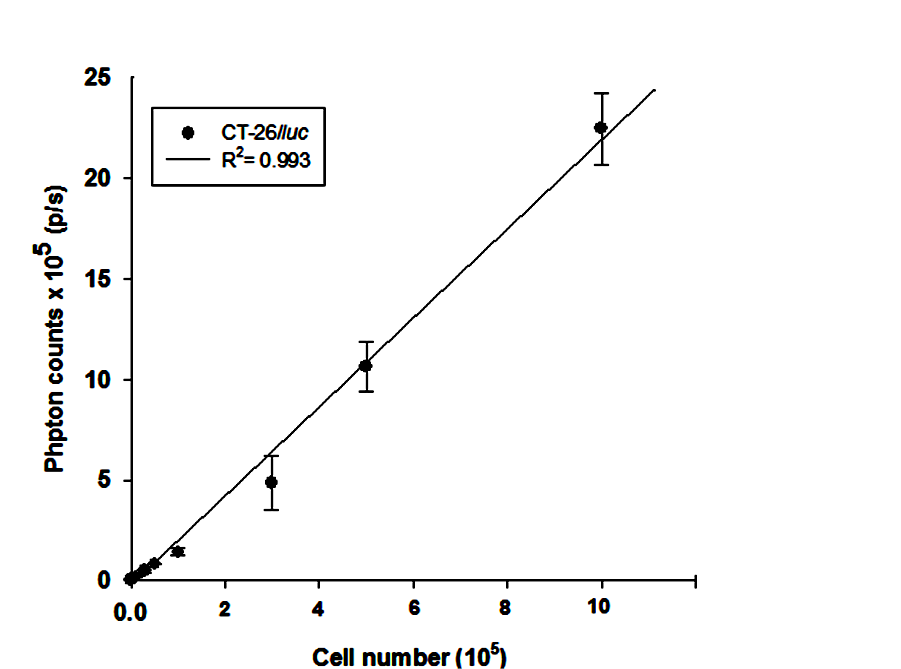


1C

0

5

10

20

40

80

1D


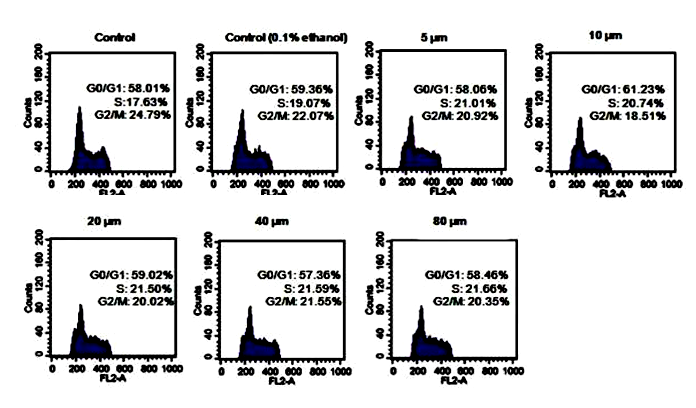

Supplement: Figure S1 — (A) The growth curves of parental CT-26 and CT-26/luc tumor cells. Td = (t-t0)×ln2/ (lnN – lnN0). The cell doubling times are 14.2 h and 14.4 h for parental CT-26 and CT26/luc cells, respectively. (B) Left: the luciferase expression in CT-26/luc cells imaged with Xenogen IVIS 50 imaging system. Right: the photon counts emitted from CT-26/luc cells is the function of the cell number with R2 = 0.993. (C) Cell viability analysis of CT26/luc cells treated with various concentrations (0, 5, 10, 20, 40, and 80 µM) of mirtazapine for 24, 48, and 72 h, and measured with MTT assay as described in the “Materials and Methods”. (D) Effect of mirtazapine on the cell cycle of CT-26/luc cells after treatment with various concentrations of mirtazapine for 24 h, and analyzed by flow cytometry. No cytotoxic effect was found. (DOC) [file pone.0038886.s001.doc]
